# Scope of Journal of Neonatal Surgery

**Published:** 2012-04-01

**Authors:** Bilal Mirza

**Affiliations:** Department of Pediatric surgery, The Children’s Hospital and the Institute of Child Health Lahore, Pakistan

The second issue of Journal of Neonatal Surgery has been successfully published. It is a matter of great pride and celebration for the editorial team. Every editor has contributed to the best of the capacity to bring forth the issue within the specified time. Professor Yogesh Kumar Sarin led us from the front; his meticulous efforts are worth a mention.

The primary scope of the journal is provision of a platform for publication of evidence-based neonatal surgery. It is the only available journal dedicated to the specialty. Neonatal surgery is often performed by pediatric surgeons in many parts of the world. Surgical neonates, the targeted proportion of the population, are entirely different with other age groups. Their body composition, requirements, immunity, pathophysiology, and response to various diseases are specific to this age group [1]. Management of surgical neonates often starts while in-utero, especially in those with congenital malformations. The decision to continue or terminate pregnancy in a case of congenital malformation is generally based on the type and severity of the malformation, parents’ will, country’s law, or religious recommendations etc. Similarly, there are other issues like management of surgical neonates in surgical neonatal intensive care unit (NICU) or medical NICU; should they be operated in operation theatre or in surgical NICU etc. Here, the role of a specialty journal as a forum for policy making and recommending cannot be ignored. In this issue, a debate on “where should the surgical neonates be nursed?” is published to help find the answer.

Often the surgical practice in neonatal population is minimally evidenced. Hall et al reviewed the literature and found that most of the surgical practice in neonatal surgery is not supported by randomized controlled trials (RCTs) [2]. The current practice is largely based on the retrospective reviews and individual preferences [2]. The better scope of the journal is to publish highly evidenced neonatal surgery as RCTs. Various categories have been developed for different kind of publications each with its own level of evidence and significance. Case reports are also published to support clinical evidence, though they are considered to represent lowest level of clinical evidence.

Electronic publishing has recently evolved as a preferred mode of publication. Print journals have also started publishing online for their continued existence [3]. This mode of publishing has given us the opportunity and freedom of space, as not the case with print journals. A great deal of manuscripts can be published at a time, without delaying the delivery of latest research to the readers. Since the launch [4], a few original articles have been published; of which majority are descriptive analyses. We are committed to publish RCTs in future; nevertheless, a limited span of the age is related to the specialty which may be the reason of more retrospective analyses than proper RCTs. The same is true for more case reports that are being published in the age group. This problem can be handled by publishing Meta-analyses of these descriptive series that might infer more concrete evidence.

The parallel scope of the journal is Continuing Professional Development (CPD) which states that everyone in any field or discipline must have up-to-date knowledge-base and skills [5]. The clinical data on various diseases and their management are dynamic and optimum therapies are continually being evolved. Therefore, it is our endeavor to publish recent literature on neonatal surgery help support the CPD. The journal also provides an opportunity to the junior surgeons and residents to learn the basic research and publication. Research papers are welcome from the junior lot. A complete section under the heading of “Face the Examiner” is especially devoted to the residents to check their basic knowledge about important neonatal surgical disorders. Similarly, “Athena’s Pages” will keep the readers abreast of latest research in the field.

The journal is in the beginning. It has been indexed with few journal-databases. Indexing with PubMed Central, PubMed, and acquisition of Impact Factor are the goals that would be achieved once the journal qualifies the recommended number of publications. Every manuscript that has been published since the launch would be indexed with all known and credulous indexing agencies. Moreover, neonatal/pediatric surgery associations and groups are invited to become our partners. We also invite conference officials to use this journal as a platform for their publications.

## Footnotes

**Source of Support:** Nil

**Conflict of Interest:** None declared

